# Diagnostic Yield of Next-Generation Sequencing for Rare Pediatric Genetic Disorders: A Single-Center Experience

**DOI:** 10.3390/medsci13020075

**Published:** 2025-06-09

**Authors:** Milena Stoyanova, Dinnar Yahya, Mari Hachmeriyan, Mariya Levkova

**Affiliations:** 1 Department of Medical Genetics, Medical University Varna, Marin Drinov Str 55, 9000 Varna, Bulgaria; 2 Laboratory of Medical Genetics, St. Marina Hospital, Hristo Smirnenski Blv 1, 9000 Varna, Bulgaria

**Keywords:** next-generation sequencing, rare disease, diagnostic yield, success rate, genetic counseling

## Abstract

**Background:** Next-generation sequencing (NGS), particularly whole-exome sequencing (WES), has become a powerful diagnostic tool for rare genetic conditions. However, its success rate varies based on the underlying genetic etiology and the population studied. **Methods**: This retrospective study evaluated the diagnostic yield of NGS in a cohort of 137 pediatric patients with suspected rare genetic disorders in Bulgaria, a setting where such testing is not reimbursed and must be self-funded. The patients underwent either WES or targeted gene panel testing based on clinical presentation, family history, and genetic evaluation. **Results**: The overall diagnostic yield was 45.99%, with WES achieving 51.25% and targeted testing achieving 38.60%. The highest yield was observed in patients presenting with both dysmorphic features and neurodevelopmental delays (62.5%), while the lowest was observed among those with isolated neurodevelopmental issues (10%). A significant portion of the identified variants (35.9%) were novel. Eight patients were diagnosed with copy number variants (CNVs) detected only through WES. **Conclusions**: Our findings illustrate the value of WES as a first-line test and highlight the impact of deep phenotyping on diagnostic success. This study also emphasizes the need for a population-specific reference genome and equal access to genomic diagnostics in all European countries.

## 1. Introduction

Rare diseases encompass a diverse range of nosological entities, with 72% having a genetic etiology [1]. Despite isolated incidences of less than 1 in 2000, approximately 1 in 15 individuals suffers from one of these conditions [1]. While our understanding of their underlying mechanisms is developing daily, shortening the diagnostic odyssey remains a challenge for current practice [2].

The diagnostic process for rare diseases typically involves a comprehensive clinical evaluation, which often includes a detailed patient history, physical examination, and histological assessment, along with various laboratory assessments, imaging studies, and genetic testing as confirmation steps. Identifying the exact genetic mutations is also crucial for determining the prognosis and treatment plan and providing effective post-test genetic counseling [3]. One significant advancement that improved the diagnostic yield was the introduction of next-generation sequencing (NGS) as a routine diagnostic method [2]. In rare diseases, including pediatric ones, the clinically available options vary between targeted gene panels, whole-exome sequencing (WES), and whole-genome sequencing (WGS). Depending on the phenotype, whether narrowed-down, pathognomonic, or non-specific, and if the diagnostic suspicion is unclear, first-tier testing can be targeted or WES. Apart from revealing single-gene variants explaining rare conditions, it also allows reverse phenotyping—retrospectively discovering unrecognized features based on genotypic findings. This additionally shortens the diagnostic odyssey, as some signs typically occur later in the evolution of a disease [4].

WES, introduced in 2009, is a popular technique that sequences the genome’s protein-coding regions, focusing on the exons. In 2010, it contributed to the recognition of a new gene responsible for a Mendelian disorder and has been further incorporated into clinical practice ever since [5]. The human exome accounts for less than 2% of the genome yet contains approximately 85% of the identified disease-related variants. This characteristic makes exome analysis a more cost-effective option than whole-genome sequencing. Consequently, exome sequencing can reveal diagnoses for various rare monogenic disorders and can identify risk variants for common and cancer-related diseases [6]. Furthermore, its lower cost is essential in countries with developing economies, such as Bulgaria, that do not reimburse patients for it through their national healthcare coverage [7]. Still, this creates a substantial access limitation for families who cannot afford it and establishes barriers during a period that promotes equity over equality.

We aim to share our experience and perspective on the diagnostic yield of NGS in a pediatric cohort of patients with suspected rare diseases. To the best of our knowledge, this is the first study to describe the diagnostic yield of NGS techniques as patient-paid methods for diagnosing rare disorders in our country. As a result, cultural and economic variations may lead to differences compared to other countries and systems. We anticipate that our data will serve as a resource for comparison and further analysis in forthcoming studies.

## 2. Materials and Methods

This retrospective study was conducted at the Laboratory of Medical Genetics at the University Hospital “St. Marina” in Varna, Bulgaria. Pediatric patients (aged ≤ 18 years) referred for genetic evaluation with a suspected genetic disorder who underwent next-generation sequencing (NGS) between 1 January 2023 and 31 December 2024 were included in this study.

A certified medical geneticist clinically assessed all patients. In accordance with institutional and ethical regulations, written informed consent was obtained from the parents or legal guardians of all participants. Clinical and demographic data were extracted from electronic medical records and genetic testing reports.

The patients were classified into four phenotypic categories based on the presence of dysmorphic features, multiple congenital anomalies, and/or neurodevelopmental delays/intellectual disabilities:

- Group 1 (Dysm/ND): Both dysmorphism/multiple congenital anomalies and a neurodevelopmental delay/intellectual disability.
- Group 2 (Dysm/MCA): Only dysmorphism/multiple congenital anomalies.
- Group 3 (ND/ID): Only a neurodevelopmental delay, an intellectual disability, or autistic features.
- Group 4 (No Dysm/ND): Neither dysmorphism nor neurodevelopmental issues. This group included individuals with other symptoms such as seizures, muscle hypotonia, frequent infections, gastrointestinal problems, and failure to thrive.

All of the patients from Groups 1, 2, and 3 had previously undergone conventional karyotyping with normal results. In addition, patients presenting with intellectual disabilities and autistic features were routinely tested for Fragile X syndrome as part of their diagnostic work-up.

Samples (peripheral blood, buccal swabs, or extracted DNA) were sent to Blueprint Genetics (Helsinki, Finland), an accredited (CLIA, CAP, and ISO 15189) external diagnostic laboratory. The patients underwent either singleton whole-exome sequencing (WES) or targeted next-generation sequencing (NGS) based on clinical indication, prior testing history, and expert clinical judgment.

Sequencing was conducted using hybridization-based capture and Illumina technology. Quality metrics ensured high coverage and reliable variant detection. Bioinformatics analysis included alignment to the GRCh37/hg19 reference genome, variant calling, annotation, and classification according to ACMG/AMP guidelines [8]. Public databases (e.g., ClinVar and gnomAD), in silico prediction tools, and the relevant literature were also consulted to support the classification of the reported variants [9,10]. The variants were categorized as pathogenic, likely pathogenic, variants of uncertain significance (VUSs), or benign. Only the pathogenic and likely pathogenic variants were included in the calculation of the diagnostic yield. The detected variants were further described by molecular type (e.g., missense, nonsense, frameshift, copy number variation (CNV), splice site, and in-frame indels), inheritance pattern, and novelty status (novel vs. reported). Descriptive statistics were used to summarize the patient characteristics and variant classifications. Chi-square tests were used to assess differences in diagnostic yield based on the sequencing method (WES vs. targeted) and the clinical phenotype group. Statistical analyses were conducted using IBM SPSS Statistics (version 26), and *p*-values < 0.05 were considered statistically significant. All numbers other than the *p*-values were rounded to one decimal place.

## 3. Results

Over a two-year period, next-generation sequencing (NGS) was performed on a cohort of 137 pediatric patients, aged from 3 days to 18 years (mean age: 5.7 years). The cohort consisted of 89 boys (65.0%) and 48 girls (35.0%).

Two sequencing approaches were utilized: whole-exome sequencing (WES) in 80 patients (58.4%) and targeted gene panel analysis in 57 patients (41.6%). Among the WES cohort, 51 (63.8%) were males and 29 (36.2%) were females, with a mean age of 5.45 years. In the targeted analysis group, 38 (66.7%) were males and 19 (33.3%) were females, with a mean age of 6.0 years.

Prior genetic investigations (multiplex ligation-dependent probe amplification (MLPA) or targeted sequencing) were conducted in 41 out of 80 WES patients (51.2%), while MLPA or polymerase chain reaction (PCR) analysis was conducted in 6 out of 57 targeted patients (10.5%).

Based on clinical presentation, the patients were categorized into four predefined phenotype groups, as described in the Materials and Methods Section. The group sizes were as follows: Group 1—56 patients (40.9%), Group 2—20 patients (14.6%), Group 3—10 patients (7.3%), and Group 4—51 patients (37.2%) (Table 1).

WES identified pathogenic or likely pathogenic variants in 41 of 80 patients, resulting in a diagnostic yield of 51.3% (Supplementary table). Targeted sequencing revealed a diagnosis in 22 of 57 patients, corresponding to a yield of 38.6% (Supplementary table). The overall diagnostic yield for both methods was 46.0% (63/137 patients).

Variants of uncertain significance were reported in 21 out of 80 WES cases (26.5%) and 8 out of 57 targeted cases (14.0%).

**Figure 1:**
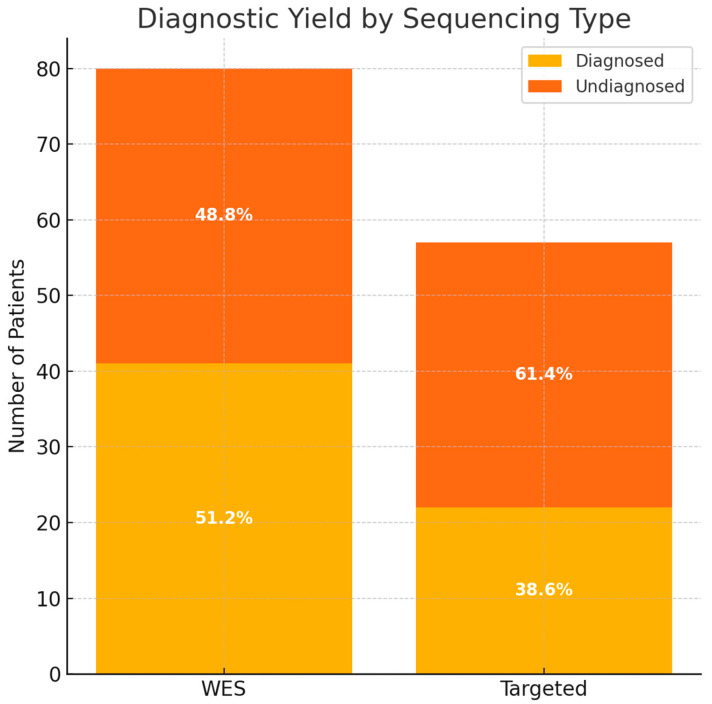
The diagnostic yield based on the type of sequencing. WES—whole-exome sequencing.

A comparative analysis showed that while the diagnostic yield was higher in the WES group (51.2%) compared to the targeted group (38.6%), this difference was not statistically significant (Chi-square test, χ^2^ = 1.67, *p* = 0.197), suggesting comparable diagnostic performance between the two approaches in this cohort (Figure 1).

**Figure 2:**
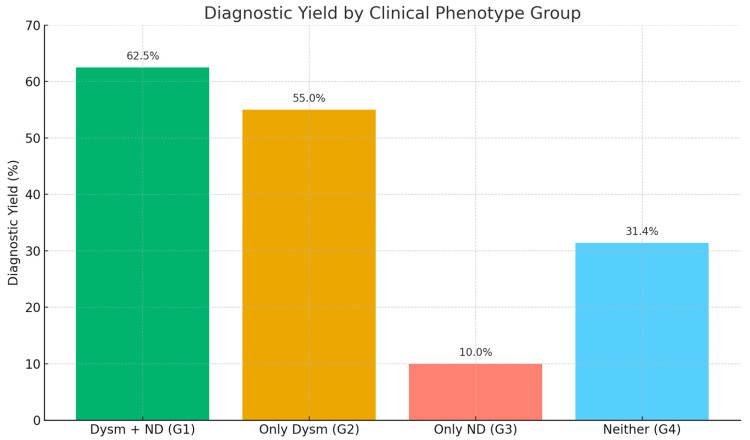
The diagnostic yield of the applied molecular genetic analyses based on the clinical phenotypes of the patients. Dysm—dysmorphism, ND—neurodevelopmental delay.

The diagnostic yield was highest among the patients with both dysmorphic features and neurodevelopmental delays (Group 1), with 35 out of 56 patients diagnosed (62.5%). Group 2 (only dysmorphism) had a yield of 55.0% (11/20), followed by Group 4 (no dysmorphism or neurodevelopmental features) with 31.4% (16/51) and Group 3 (only neurodevelopmental delays) with 10.0% (1/10). The comparison revealed a statistically significant difference among the groups (Chi-square test, χ^2^ = 16.8, *p* = 0.0009), indicating a strong association between the clinical phenotype and the likelihood of obtaining a genetic diagnosis. The patients exhibiting both structural anomalies and developmental concerns demonstrated the highest level of diagnostic success (Figure 2).

**Figure 3:**
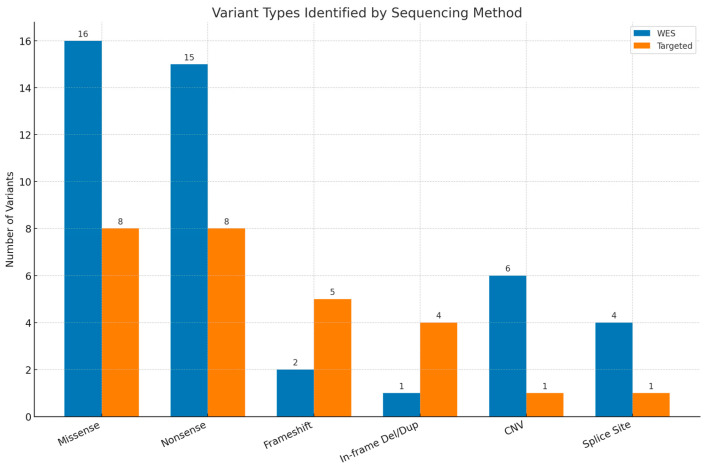
Types of variants reported by whole-exome sequencing (WES) and targeted testing. Del—deletion, dup—duplication, CNV—copy number variant.

Among the 63 patients with pathogenic or likely pathogenic variants, 56 (88.9%) were diagnosed with Single-Nucleotide Variants (SNVs)/insertions or deletions (INDELs) and 7 (11.1%) were diagnosed with CNVs. The most frequently observed variants were missense mutations, identified in 16 WES cases and 8 targeted cases, followed by nonsense (stop-gained) variants (15 WES cases and 8 targeted cases). Figure 3 presents the distribution of the detected variants.

Of the 56 patients with pathogenic or likely pathogenic SNVs, 36 had autosomal dominant (AD) disorders, 16 had autosomal recessive (AR) disorders, and 4 had X-linked conditions. Additionally, seven diagnoses were attributed to CNVs.

At the time of reporting, 35.9% of the identified variants—16 WES cases and 7 targeted sequencing cases—were novel and had not been reported in public databases or the literature. Furthermore, 60.9% of the variants (29 WES cases and 10 targeted cases) were absent from the gnomAD population database [10].

Of the patients who received a positive molecular diagnosis, all (100%) were offered post-test genetic counseling. In several families, this enabled informed reproductive decision-making, risk assessment for relatives, and clarification of recurrence risks. Due to a lack of systematic long-term follow-up and clinical documentation, we were unable to quantify the proportion of cases where diagnosis prevented invasive investigations or led to early interventions such as pre-symptomatic sibling testing.

Overall, next-generation sequencing demonstrated a clinically meaningful diagnostic yield, especially in patients presenting with both structural and developmental phenotypes. WES yielded a higher diagnostic rate, though this difference was not statistically significant, and a diverse range of variant types and inheritance patterns were identified across the cohort.

## 4. Discussion

Over the past few years, WES has become the standard diagnostic tool for evaluating patients with dysmorphic features, congenital anomalies, and intellectual disabilities. Its clinical utility was further confirmed in 2021, when the American College of Medical Genetics and Genomics recommended WES as a first- or second-tier diagnostic test for individuals presenting with such symptoms [8].

Despite high public expectations, no molecular genetic test has achieved a diagnostic yield approaching 100%. For instance, the Deciphering Developmental Disorders (DDD) study, which included 13,449 individuals and applied WES testing, reported a diagnostic success rate of 41% [11]. In our cohort, the diagnostic yield was slightly higher (46.0% for both applied types of testing) than previously reported figures, which may be attributed to our smaller sample size and the deep phenotyping of all participants. WGS, which emerges as a testing option in patients with a negative result from WES, does not have a significantly higher diagnostic capacity. A large meta-analysis on pediatric patients with suspected genetic disorders reports success rates of 38.6% for WGS and 37.8% for WES [12]. WGS can further increase the diagnostic success rate by enabling the detection of non-coding variants and large insertions or deletions. Unlike WES, WGS provides coverage of non-coding regions, including regulatory and deep intronic areas that may play a role in disease pathogenesis. As such, WGS can significantly enhance the overall diagnostic yield, particularly in cases where WES results are inconclusive [13].

Our genetic counseling unit is the only one serving the eastern region of our country, providing approximately 200 consultations annually for children with suspected rare genetic conditions. In Bulgaria, full reimbursement for genetic testing is only available for individuals under 18 years of age and only if the testing is performed during hospitalization [7]. Consequently, many families bear the cost of genetic testing themselves, often selecting their testing strategy based on financial considerations. Because WES remains more expensive than targeted testing, we recommend the latter when clinical features suggest a specific genetic disorder and a positive result is anticipated even with a more limited testing approach. For this reason, our study included patients who underwent both WES and targeted genetic analyses.

Our results indicate that the diagnostic yield of genetic testing is strongly influenced by the clinical presentation of the patients. The highest success rate was observed in the patients from Group 1, who presented with both dysmorphic features and neurodevelopmental delays—in this group, 62.5% (35/56) received a genetic diagnosis. This suggests that the presence of both structural anomalies and developmental symptoms significantly increases the likelihood of detecting an underlying genetic disorder.

Also, the patients from Group 2, who only had dysmorphic features, showed a relatively high yield (55.0%, 11/20), illustrating that structural anomalies alone can be potential markers for underlying genetic conditions.

On the other hand, the lowest diagnostic yield of the NGS testing in our study was registered for Group 3 (patients with only neurodevelopmental delays): 10.0% (1/10). This could be explained by the greater clinical and genetic heterogeneity associated with isolated neurodevelopmental symptoms [14]. This was also reported by other research groups—for example children with isolated autism spectrum disorder had a greater chance of receiving negative WES results [15].

The statistically significant difference in the diagnostic yield among the groups underscores the importance of deep phenotyping when choosing specific genetic testing strategies. These findings align with prior studies suggesting that the diagnostic success of next-generation sequencing increases with the complexity and specificity of the clinical phenotype [16].

Our phenotypic categorization of patients into four distinct groups based on neurodevelopmental features, dysmorphic features, and associated clinical findings provides a practical framework that could guide genetic testing strategies. Although our study was not initially designed to establish formal diagnostic criteria, the observed correlation between specific phenotypic patterns and the diagnostic yield suggests that such categorization could serve as a valuable tool for prioritizing patients for WES or targeted NGS testing. For example, the patients in Groups 1 and 2 demonstrated a higher diagnostic yield, indicating that early NGS testing may be more justified in these subgroups. Developing a clinical decision-making pathway based on these phenotypic categories may enhance the efficiency and cost-effectiveness of genetic investigations, particularly in settings with limited access to comprehensive genomic testing.

Our study found that a significant proportion of the reported variants were novel, highlighting the limitations of current genomic databases and the need for broader representation. The commonly used human reference genome, based on sequencing data from only approximately 20 individuals, lacks diversity and may obstruct accurate variant interpretation, especially in underrepresented populations [17]. Without broader inclusion, many population-specific or rare variants may be misclassified as VUSs, potentially delaying or preventing accurate diagnosis. In our cohort VUSs were reported in 21 out of 80 WES cases (26.5%). A more inclusive pangenome reference, reflecting global genetic diversity, could lower the number of these uncertain findings and improve diagnostic accuracy [17]. This was also noted by another research group, who claim that the diagnostic yield depends on the studied population [18].

Consanguinity further influences the variant landscape. In our country, consanguineous marriages are relatively common among minority populations, increasing the likelihood of homozygous pathogenic variants. The Roma minority, in particular, is a genetic isolate with frequent consanguinity that has a distinct distribution of private pathogenic variants [19]. Better characterization of these population-specific alleles is critical for improving diagnostic precision in these groups.

The majority of the pathogenic CNVs in our cohort (six out of seven cases) were identified through WES, underscoring its value as a first-line diagnostic tool. In contrast, only one case was diagnosed via targeted testing, highlighting the relatively limited yield of more focused approaches [8]. However, in our country, WES is not reimbursed and is paid out of pocket by patients, making its higher cost a significant barrier to access. As a result, WES was performed as a second-tier test for nearly half of the patients in the WES group, often following less expensive genetic testing. This was also the reason that we did not have any WES trios in our cohort.

Several limitations should be considered. The relatively small sample size may limit the generalizability of our findings. The lack of trio-based WES, due to financial constraints, hindered segregation analysis in some cases. In addition, financial barriers to genetic testing in Bulgaria may have introduced a degree of selection bias, as families with greater financial means were more likely to access testing. Also, as a single-center study, our results may not fully represent the broader genetic landscape in this country. Lastly, the absence of a national electronic medical record system makes it impossible to systematically follow up with patients who have received a positive genetic result and to assess the implications of these findings for subsequent therapy adjustments, surveillance strategies, or reproductive counseling. There is a pressing need to establish a national rare-disease registry, which would enable clinicians to track clinical management outcomes and support future research in this area.

Despite these limitations, our findings highlight the clinical value of NGS, particularly WES, in the diagnostic evaluation of children with suspected genetic disorders and underscore the need for wider access to genomic services in Bulgaria.

## 5. Conclusions

This is the first study to evaluate the diagnostic yield of NGS in pediatric patients with rare genetic disorders in Bulgaria. Our findings demonstrate a diagnostic yield comparable to previously published data, with a higher success rate in patients presenting with both dysmorphic features and neurodevelopmental disorders. The high number of novel variants highlights the need for a population-specific reference genome to improve diagnostic accuracy. Importantly, our proposed phenotypic categorization may aid clinicians in selecting between targeted gene panels and whole-exome sequencing, offering a more efficient and cost-effective approach to genetic testing.

## Figures and Tables

**Table 1:** The patient categories in the present research. Dysm—dysmorphism, ND—neurodevelopmental delay, MCA—multiple congenital anomalies, ID—intellectual disability.

Characteristic	WES Group	Targeted Group	Overall
Total patients (%)	80 (58.4%)	57 (41.6%)	137
Mean age (years)	5.45	6	5.72
Male patients (%)	51 (63.8%)	38 (66.7%)	89 (65.0%)
Female patients (%)	29 (36.3%)	19 (33.3%)	58 (35.0%)
Prior genetic investigations (%)	41 (51.3%)	6 (10.5%)	48 (35.0%)
Group 1 (Dysm/ND) (%)	48 (60.0%)	8 (14.0%)	56 (40.9%)
Group 2 (Dysm/MCA) (%)	10 (12.5%)	10 (17.5%)	20 (14.6%)
Group 3 (ND/ID) (%)	9 (11.3%)	1 (1.8%)	10 (7.3%)
Group 4 (No Dysm/ND) (%)	13 (16.3%)	38 (66.7%)	51 (37.2%)

## Data Availability

The authors confirm that the data supporting the findings of this study are available within this article.

## Supplementary Materials

The following supporting information can be downloaded at: https://www.mdpi.com/article/10.3390/medsci13020075/s1, Supplementary table with information on the reported findings.

## Abbreviations

The following abbreviations are used in this manuscript:

ACMG/AMP	American College of Medical Genetics and Genomics/Association for Molecular Pathology
AD	autosomal dominant
AR	autosomal recessive
CNV	copy number variation
Del	deletion
DDD	Deciphering Developmental Disorders
Dup	duplication
Dysm	dysmorphism
ID	intellectual disability
INDEL	insertion or deletion
MCA	multiple congenital anomalies
MLPA	multiplex ligation-dependent probe amplification
ND	neurodevelopmental delay
NGS	next-generation sequencing
SNV	Single-Nucleotide Variant
VUS	variant of uncertain significance
WES	whole-exome sequencing
